# Evaluation of the effectiveness and safety of icariin in the treatment of knee osteoarthritis: A protocol for a systematic review and meta-analysis

**DOI:** 10.1097/MD.0000000000028277

**Published:** 2021-12-17

**Authors:** Lang Liu, Changwei Zhao, Shuang Zhao, Hanxun Xu, Zeyu Peng, Binghua Zhang, Wenjun Cai, Yifang Mo, Wenhai Zhao

**Affiliations:** a Changchun University of Chinese Medicine, 1035 Boshuo Road, Changchun, Jilin, China; b Affiliated Hospital of Changchun University of Chinese Medicine, 1478 Gongnong Road, Changchun, Jilin, China; c The Third Affiliated Hospital of Changchun University of Chinese Medicine, 1643 Jingyue Street, Changchun, Jilin, China.

**Keywords:** icariin, knee osteoarthritis, meta-analysis, protocol, systematic review

## Abstract

**Background:**

Knee osteoarthritis (KOA) is a chronic degenerative disease involving cartilage and surrounding tissues. It causes a huge burden to social and medical resources and seriously affects people's living and working ability. In recent years, people have become increasingly interested in the application of Chinese medicine monomers to treat KOA. Among them, icariin plays an important role in the clinical treatment of KOA. Therefore, to evaluate the effectiveness and safety of icariin in the treatment of KOA, we conducted this study to provide a new basis for the clinical treatment of KOA.

**Methods:**

We propose a systematic search of the PubMed, Embase, Web of Science, Cochrane Library, China National Knowledge Infrastructure, Wanfang, and China Biomedical databases for all randomized controlled trials examining the use of icariin in the treatment of KOA patients up to October 20, 2021. The screening and data extraction processes will be performed independently by 2 researchers. We will use the Cochrane risk bias assessment tool to evaluate the quality of the studies that met the inclusion criteria. The data will be statistically analyzed using RevMan5.3 software.

**Result:**

This study will provide high-quality evidence for the effectiveness and safety of icariin in the treatment of KOA.

**Conclusion:**

The purpose of this study was to explore the efficacy of icariin in the treatment of KOA and to provide clinicians and patients with new treatment strategies.

**INPLASY registration number:**

INPLASY2021110015.

## 1 Introduction

Knee osteoarthritis (KOA) is the most common chronic progressive disease in the world and one of the main causes of physical disabilities.^[1,2]^ The knee is the joint with the highest incidence of osteoarthritis, and the degeneration of this joint can seriously affect people's daily lives.^[3,4]^ An epidemiological survey found that the global prevalence of KOA is 16% among people over 15 years old and 22.9% among people over 40 years old. As of 2020, there were approximately 654.1 million people suffering from KOA worldwide. The incidence of KOA varies greatly between different countries and increases with age.^[5]^

Although the incidence of KOA is increasing year by year, its pathogenesis is still not completely clear.^[6]^ Recent studies have reported additional factors leading to the development of KOA, such as external mechanical load (including obesity), joint trauma, metabolism, and genetics.^[7,8]^ The current treatments for KOA include drug therapy, physical therapy, exercise, intra-articular injection therapy, cognitive behavioral therapy, cell therapy, and even surgery.^[9,10]^ Non-steroidal anti-inflammatory drugs (NSAIDs) are the first-line drugs for KOA and can effectively relieve pain. However, for elderly and frail patients with osteoarthritis, long-term use of oral NSAIDs can affect the gastric mucosa and cause adverse reactions such as gastric ulcers and gastric bleeding.^[11]^ Therefore, it is important to find a drug that can replace oral NSAIDs in the treatment of KOA. In recent years, an increasing number of scientific researchers have turned their attention toward examining the efficacy and mechanism of using Chinese herbal extracts to treat KOA.^[12]^

Icariin is a flavonoid compound isolated from the traditional Chinese medicine Epimedium. It is the main biologically active ingredient in Epimedium. It has a long history of clinical treatment of bone and joint diseases in China.^[13]^ Many in vitro and animal studies have found that icariin has a positive effect in the treatment of KOA, which can inhibit bone resorption while inducing bone formation.^[14]^ Animal experiments have confirmed that icariin can promote the differentiation of Sprague–Dawley rats (6 months old) bone marrow mesenchymal stem cells (BMSCs) into osteoblasts.^[15]^

In summary, an increasing number of studies have reported the potential value of icariin in the treatment of KOA, but its effectiveness and safety need more clinical evidence for verification. Therefore, this study proposes a systematic review of icariin for the treatment of patients with KOA.

## 2 Methods and analysis

### 2.1 Information sources and search strategy

This protocol describes a systematic review and meta-analysis of previously published studies, so ethical approval is not required. This study was designed in strict accordance with the 2015 Preferred Reporting Items for Systematic Reviews and Meta-Analyses Protocols (PRISMA-P).^[16]^ The prospective registration was approved by the International Platform of Registered Systematic Review and Meta-analysis Protocols (https://inplasy.com/inplasy-2021-11-0015/) under registration number inplasy2021110015. We will search 4 foreign language electronic databases, including PubMed, Embase, Web of Science, and Cochrane Library, and 3 Chinese literature databases, including the China National Knowledge Infrastructure Database, Wanfang Database, and China Biomedical Database. Two researchers will screen randomized controlled trials (RCTs) examining the use of icariin in the treatment of KOA, and the main search terms will be “icariin” and “KOA”.

### 2.2 Included and excluded criteria

#### 2.2.1 Type of studies

Only RCTs using icariin in the treatment of KOA will be included. Non-RCTs, review papers, qualitative studies, and documents with incomplete data will be excluded.

#### 2.2.2 Participants

The participants will be patients who have been diagnosed with KOA according to the criteria established by the American College of Rheumatology without restrictions based on sex, age, race, time of onset, and course of disease.

#### 2.2.3 Interventions

The experimental group will comprise individuals who used icariin as an intervention in RCTs. There will be no restriction regarding the method of administration, time, dosage, or cycle during intervention. The control group will comprise individuals who were treated with placebo or other alternative drugs.

#### 2.2.4 Outcome measures

The primary outcome measures will be the Western Ontario and McMaster Universities Osteoarthritis Index, visual analog scale, and total effective rate.

The secondary outcome measures will be the Short-Form 36 and adverse reactions.

### 2.3 Literature screening and data extraction

Literature screening and data extraction will be independently performed by 2 skilled researchers (LL and SZ). Articles retrieved from the literature search will be managed by Endnote X9 software (Clarivate Analytics). First, the 2 researchers (LL and SZ) will independently read the title and abstract of each article and conduct a preliminary screening to exclude duplicate studies. Then, they will read the full text of each article further to determine which articles meet the inclusion criteria. Finally, they will extract data from the articles that meet the inclusion criteria. The extracted information will include title, author, publication time, number of cases, age, intervention measures, outcome, and risk assessment of bias. The data will also be cross-checked. If there is a disagreement, the third researcher (WZ) will be consulted.

### 2.4 Risk of bias in assessment

We will use the risk of bias assessment tool recommended by Cochrane System Reviewer Manual 5.1.0 to evaluate the quality of the included RCTs. Two researchers (BZ and ZP) will perform the analysis, and then, their evaluations will be cross-checked. We will evaluate the following aspects: random sequence generation, allocation concealment, participants, personnel, blinding of result evaluation, completeness of result data, selective reporting, and other deviations. The assessment results will be divided into 3 levels: low risk, high risk, and uncertain risk. Disagreements in the evaluation of the quality of the literature will be resolved by discussion or judgement from an expert (WZ).

### 2.5 Statistical analysis

Meta-analysis of the main outcome measures will be performed using RevMan 5.3 software (Cochrane Collaboration). In this study, odds ratios will be used as effect measures for binary variables. For measurement data with the same unit and measurement method, the mean difference will be used along with the 95% confidence interval. *P* < .05 will indicate significant differences. The χ2 test will be used to examine heterogeneity. If *P* > .05 and I^2^ ≤ 50%, then a fixed effects model will be used for meta-analysis; otherwise, a random effects model will be used for meta-analysis. Obvious clinical heterogeneity will be addressed by subgroup analysis, sensitivity analysis, or descriptive analysis.

### 2.6 Subgroup analysis and sensitivity analysis

If the heterogeneity between the studies is substantially large, subgroup analysis will be carried out. Subgroup analysis can be performed according to the patient's age, race, intervention, type of treatment, and duration. Sensitivity analysis is used to assess the stability and reliability of meta-analysis results. We will perform sensitivity analysis or descriptive analysis by eliminating studies one at a time to assess the stability of the results.

### 2.7 Reporting biases assessment

If more than 10 articles are included in a certain outcome index, an inverted funnel chart will be used to analyze whether there is evidence of publication bias.

### 2.8 Ethics and dissemination

The data included in this study will be derived from published articles, so ethical approval is not required.

## 3 Discussion

With the accelerating process of population aging, KOA has become an important disease affecting public health. The pathological changes of KOA involve articular cartilage, synovium, subchondral bone, ligaments, adipose tissue, and meniscus. Its pathogenesis is complicated, and it seriously reduces the patient's quality of life.^[17,18]^ Inflammation plays an important role in the pathogenesis of KOA.^[19]^

The protective effect of icariin on KOA has multiple molecular mechanisms. Animal experiments show that icariin can reduce inflammation and promote angiogenesis and has the potential to promote wound healing in diabetic rats.^[20]^ Previous studies have found that icariin can inhibit the adipogenic differentiation of bone marrow mesenchymal stem cells by downregulating the expression of peroxisome proliferator activated receptor γ.^[21]^ Bone growth and development require adequate blood supply. Icariin can promote angiogenesis by stimulating the migration and proliferation of endothelial cells in the body.^[22]^

As people pay more attention to healthy lifestyles, an increasing number of people have chosen to use traditional Chinese medicine interventions to treat KOA. Currently, there are few RCTs examining the use of icariin in the treatment of KOA. Therefore, this study will systematically evaluate the effectiveness and safety of icariin in the treatment of KOA and provide evidence-based medicine guidance for icariin in the treatment of KOA.

## Author contributions

**Conceptualization:** Lang Liu, Wenjun Cai.

**Data curation:** Lang Liu, Shuang Zhao, Zeyu Peng.

**Formal analysis:** Lang Liu, Shuang Zhao, Hanxun Xu.

**Funding acquisition:** Changwei Zhao, Wenhai Zhao.

**Investigation:** Hanxun Xu, Wenjun Cai.

**Methodology:** Binghua Zhang, Yifang Mo.

**Project administration:** Changwei Zhao, Wenhai Zhao.

**Supervision:** Changwei Zhao, Wenhai Zhao.

**Writing – original draft:** Lang Liu.

**Writing – review & editing:** Lang Liu, Changwei Zhao, Wenhai Zhao.
